# Robotic endoscopic cardiac surgery in the reoperative patient: Is it feasible?

**DOI:** 10.1016/j.xjse.2025.100079

**Published:** 2025-09-17

**Authors:** Yazan N. AlJamal, Sarah Nisivaco, Husam H. Balkhy

**Affiliations:** Department of Cardiovascular Surgery, University of Chicago, Chicago, Ill

**Keywords:** robotic cardiac surgery, redo cardiac surgery, minimally invasive surgery, totally endoscopic surgery, coronary artery bypass grafting, mitral valve surgery, aortic valve replacement, reoperative cardiac surgery, robotic TECAB, robotic mitral repair

## Abstract

**Objective:**

Robotic cardiac surgery is increasingly adopted in the United States but remains a relative contraindication in reoperative settings for many programs. We hypothesize that in experienced hands, robotic approaches offer significant advantages in redo cardiac surgery by avoiding graft and cardiac injury during sternal reentry. We present our single-center experience with robotic redo endoscopic cardiac surgery, including midterm outcomes up to 10 years.

**Methods:**

A retrospective review of 1834 robotic cardiac surgeries from 2013 to 2024 was performed. Of these, 105 patients (6%) with previous cardiac surgery were included. Patients underwent either epicardial (n = 45) or intracardiac (n = 60) procedures. Preoperative characteristics, intraoperative and postoperative outcomes, and midterm survival were analyzed.

**Results:**

In the intracardiac group, procedures included mitral valve (n = 49), aortic valve (n = 4), tricuspid valve (n = 3), cryomaze (n = 2), myxoma (n = 1), and atrial septal defect (n = 1). Epicardial procedures included totally endoscopic coronary artery bypass (n = 31), pericardiectomy (n = 4), left ventricular leads (n = 7), and others (n = 3). The mean Society of Thoracic Surgeons score was 4.0%. Thirty-day mortality was 1.9% (observed to expected ratio 0.5), and hospital mortality was 1.9%. Conversion to sternotomy occurred in 2 cases (1.9%). Intensive care unit and hospital length of stay averaged 1.6 and 3.2 days, respectively. At a mean follow-up of 37.5 months, all-cause mortality was 15%, with 6.6% cardiac-related mortality.

**Conclusions:**

Robotic endoscopic cardiac surgery is feasible and safe in selected reoperative patients. Despite the challenges of reoperation, excellent early outcomes and acceptable midterm survival were achieved. These procedures can be successfully integrated into comprehensive robotic programs to expand minimally invasive options for complex cardiac reoperations.

**Figure:**
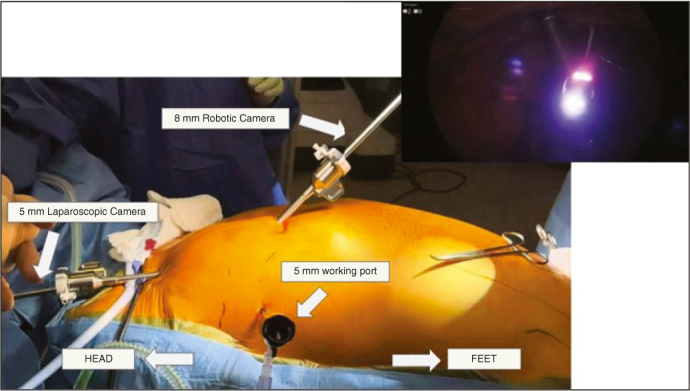
Robotic mitral valve surgery after sternotomy with right chest port placement.

**Central Message**

Robotic redo totally endoscopic coronary and mitral valve surgery is feasible and safe in selected patients, with low 30-day mortality and acceptable midterm survival.

**Perspective**

Redo robotic totally endoscopic cardiac surgery serves as a viable alternative in reoperative intracardiac and epicardial surgery in selected patients.

Reoperative cardiac surgery has traditionally been associated with significant challenges as a result of increased risks of morbidity and mortality associated with sternal re-entry. The risks include injury to critical vascular and cardiac anatomy (innominate vein, ascending aorta, right atrium and ventricle) that lie directly behind the sternum; injury to adjacent cardiac and lung structures during the adhesiolysis; inadequate exposure such as mitral valve surgery in the redo setting; and finally graft injury in patients with previous coronary bypass surgery and patent bypass grafts.^1^, ^2^, ^3^ Conventional sternotomy-based reoperations are associated with operative mortality rates ranging from 4% to 7%, which are markedly greater than those for primary cardiac surgeries.^4^, ^5^, ^6^, ^7^ Consequently, alternative approaches such as minimally invasive surgeries have been explored to mitigate these risks.^8^^,^^9^

Robotic cardiac surgery offers several advantages, including superior visualization (10 × magnification), precision in adhesiolysis, and minimized trauma to surrounding structures. Compared with minimally invasive approaches conducted by direct vision through a minithoracotomy, the endoscopic view afforded by the robotic scope offers a wide panoramic appreciation of the anatomy. Early reports demonstrate that robotic systems facilitate meticulous dissection and enable complex intracardiac and epicardial procedures with favorable outcomes.^10^, ^11^, ^12^^,^^13^

In cases of mitral valve surgery or coronary revascularization after previous sternotomy, robotic surgery has shown promise in reducing blood loss, hospital stay, and recovery times compared with traditional approaches.^11^^,^^12^^,^^17^ For coronary reoperations, alternatives like the use of the right gastroepiploic artery via subxiphoid or transdiaphragmatic approaches have been explored; however, these techniques are often limited by anatomical constraints and previous abdominal surgeries.^14^, ^15^, ^16^ Robotic-assisted totally endoscopic coronary artery bypass (TECAB) further pushes the boundaries by enabling revascularization without sternotomy or cardiopulmonary bypass and the harvesting of a frequently previously unused right internal thoracic artery conduit.^10^^,^^13^^,^^17^

Since initiating the robotic cardiac surgery program at our current institution in 2013, we hypothesized that reoperative robotic approaches could overcome the limitations of traditional sternotomy while maintaining surgical safety and efficacy.^13^ Our institution's experience spans a wide spectrum of robotic cardiac procedures, emphasizing the totally endoscopic approach with increasing application to reoperations. This study aims to evaluate the feasibility, safety, and outcomes of robotic endoscopic cardiac surgery in patients with previous cardiac operations over a decade of institutional data.

## Methods

### Study Design and Patient Population

This retrospective, single-center study was conducted at University of Chicago and included all patients undergoing robotic endoscopic cardiac surgery between July 2013 and April 2024. Institutional review board approval was obtained for this study (institutional review board #18-0742; approval date April 28, 2020). Given the retrospective nature of the study, the need for individual informed consent was waived. A total of 1834 patients underwent robotic cardiac procedures during the study period, of whom 105 patients (6%) had a history of previous cardiac surgery and constitute the focus of this review. Data collection included demographics, comorbidities, surgical details, and outcomes, with follow-up extending up to 10 years. Redo robotic cardiac cases were initiated at the University of Chicago after early institutional success with primary robotic procedures. Although the program formally began in 2013, the primary surgeon (H.H.B.) had previous extensive experience in robotic cardiac surgery dating back to 2006, facilitating early integration of complex reoperative cases into the program.

Inclusion criteria included all adult patients (age >18 years) with a history of previous cardiac surgery who underwent robotic endoscopic cardiac procedures between 2013 and 2024. Patients undergoing primary operations, nonendoscopic, or hybrid procedures were excluded. The decision to pursue a robotic approach was determined by patient anatomy, previous surgical history, and team experience, which introduces a degree of selection bias inherent to this single-center retrospective study.

### Patient Preparation and Operative Setup

Patients were positioned supine with appropriate lateral chest elevation depending on the planned surgical access (right for intracardiac or left for epicardial procedures). A single-lumen tube with bronchial blocker was used for one-lung ventilation in all cases. Defibrillator pads were placed prophylactically. In intracardiac (on-pump) procedures, the femoral artery and vein were cannulated under ultrasound guidance or using a small cut-down incision. Epicardial (off-pump) procedures required groin preparation for potential cannulation.

### Port Placement and Adhesiolysis

Initial thoracoscopic access was established through a 5-mm port placed away from previous scars in the second or fourth intercostal space at the anterior axillary or midclavicular line. A blunt needle with CO_2_ insufflation was used to create a controlled pneumothorax. The thoracoscope was introduced to evaluate adhesions, and subsequent robotic ports (camera and robotic arms) were placed under direct vision. Adhesiolysis was performed meticulously with low-energy electrocautery to clear the surgical field while minimizing tissue trauma. Robotic ports were strategically placed to optimize access to the target anatomy and avoid previous surgical sites. The surgical robot was docked from the appropriate side.

### Surgical Technique

All procedures were performed by an experienced robotic surgical team using the da Vinci Si or Xi surgical system (Intuitive Surgical) (Video 1). The approach and conduct of each procedure were tailored to the patient's anatomy and surgical requirements.

• Epicardial (off-pump) procedures: These included TECAB, coronary unroofing, pericardiectomy, and epicardial lead placement. Dissection was performed to expose target structures while preserving phrenic nerve integrity. Patent bypass grafts were identified and protected. Anastomoses in TECAB were completed using either conventional sutures or specialized anastomotic devices.
• Intracardiac (on-pump) procedures: Cardiopulmonary bypass was initiated via femoral or axillary artery cannulation. The procedure was performed either under ventricular fibrillatory arrest or aortic occlusion with antegrade cardioplegia achieving diastolic cardiac arrest. Aortic occlusion with antegrade cardioplegia was achieved using the IntraClude balloon (Edwards Lifesciences) or a transthoracic clamp. Hypothermic ventricular fibrillatory arrest was used in patients with intact aortic valve and normal ventricular function in the presence of a patent internal thoracic artery graft or when no aortic manipulation was desired. Our hypothermia ventricular fibrillatory arrest approach was discussed in a previous publication.^18^ Procedures included mitral valve repair/replacement, aortic valve replacement, atrial septal defect closure, and cryomaze ablation. Cardiac structures were accessed with minimal dissection and after establishing CPB to reduce injury.

The pericardium was routinely closed loosely with running sutures to prevent adhesion formation. When the right internal thoracic artery (RITA) was used for redo TECAB, it was routed beneath the innominate vein and covered with the anterior mediastinal fat pad to protect against future re-entry injuries. Lung surfaces dissected during adhesiolysis were treated with Progel (NeoMend) to reduce air leaks. Patients were extubated as early as possible to expedite recovery.

### Outcome Measures

The primary outcome was 30-day mortality, defined as death occurring within 30 days of the index operation, regardless of discharge status. In addition to 30-day mortality, midterm mortality outcomes were analyzed, including survival up to 10 years. Cardiac-related mortality was defined as death resulting from heart failure, myocardial infarction, fatal arrhythmia, or cardiac surgical complications. Cause of death was determined through electronic medical record review and/or direct communication with the patient's family. When the cause of death was unknown it was considered a cardiac mortality. The observed to expected ratio for 30-day mortality was calculated using only patients who underwent coronary artery bypass or intracardiac valve procedures. Patients undergoing left ventricular lead placement or pericardiectomy were excluded from this calculation.

Secondary outcomes included intensive care unit (ICU) and hospital length of stay, postoperative complications such as reoperation for bleeding, atrial fibrillation, pneumonia, myocardial infarction, stroke, acute kidney injury, and prolonged ventilation (defined as >24 hours). Pump and crossclamp were not used in all cases because a significant number were completed off pump; therefore, operative time was used as a consistent surrogate for intraoperative complexity. All patients were scheduled for structured follow-up within 30 days of discharge, conducted either in person or via telemedicine for patients residing out of town. Midterm outcomes were obtained through a combination of electronic medical record review and direct telephone contact with patients or their families. Using this methodology, follow-up completeness exceeded 95% at the time of analysis.

### Statistical Analysis

Continuous variables were presented as mean ± standard deviation or median (interquartile range) and analyzed using the Student *t* test or Mann-Whitney *U* test, as appropriate. Categorical variables were expressed as percentages and analyzed using the χ^2^ test or Fisher exact test. Statistical analyses were performed using SPSS, version 24 (IBM Corp).

## Results

### Surgical Procedure Distribution

A total of 105 patients underwent robotic redo cardiac procedures, with 45 patients (42.8%) in the epicardial group and 60 patients (57.2%) in the intracardiac group. The distribution of procedures performed in each group is summarized in Table 1.

**Table 1:** The distribution of procedures performed in the intracardiac and epicardial groups

Procedure	n
Intracardiac group	
Mitral valve surgery	49
Aortic valve surgery	4
Tricuspid valve surgery	3
Atrial septal defect closure	2
Cardiac mass excision	1
Epicardial lead placement	1
Total	60
Epicardial group	
TECAB	31
Pericardiectomy	4
LV epicardial lead placement/exchange	7
Robotic VT ablation	1
RV biopsy	1
Robotic unroofing myocardial bridge	1
Total	45

*TECAB*, Totally endoscopic coronary artery bypass; *LV*, left ventricular; *VT*, ventricular tachycardia; *RV*, right ventricular.

### Patient Demographics and Preoperative Characteristics

The mean age was 64.7 years in the epicardial group and 64.2 years in the intracardiac group. There were 31 male patients (69%) in the epicardial group and 41 (68%) in the intracardiac group. The mean BMI was 28.9 kg/m^2^ in the epicardial group and 27.3 kg/m^2^ in the intracardiac group.

Among preoperative conditions, hypertension and diabetes mellitus were present in 38 (84.4%) and 34 (75.6%) of epicardial cases and 43 (71.6%) and 32 (53.3%) of intracardiac cases. Atrial dysrhythmia was reported in 9 (20%) of epicardial cases and 27 (45%) of intracardiac cases. Chronic obstructive pulmonary disease was present in 6 (13.3%) of epicardial cases and 10 (16.7%) of intracardiac cases. The mean creatinine level was 1.64 mg/dL in the epicardial group and 1.3 mg/dL in the intracardiac group.

The mean ejection fraction was 44.9% in the epicardial group and 52.27% in the intracardiac group. Eighteen (40%) of the patients in the epicardial and 5 (8.3%) of the intracardiac group had an ejection fraction less than 40%. Eleven (24.4%) in the epicardial group and 5 (8.3%) of the patients in the intracardiac group had a previous permanent pacemaker. The mean preoperative Society of Thoracic Surgeons score was 4.6% in the epicardial group and 3.6% in the intracardiac group (Table 2).

**Table 2:** Patient demographics and preoperative characteristics

Variables	Epicardial group (n = 45)	Intracardiac group (n = 60)
Mean age, y, mean ± SD	64.7 ± 12.2	64.2 ± 12.4
Male, n (%)	31 (69%)	41 (68%)
Mean BMI, kg/m^2^, mean ± SD	28.9 ± 5.64	27.3 ± 5
Hypertension, n (%)	38 (84.4%)	43 (71.6%)
Diabetes mellitus, n (%)	34 (75.6%)	32 (53.3%)
Atrial dysrhythmia, n (%)	9 (20%)	27 (45%)
Previous CVA, n (%)	4 (8.9%)	13 (21.6%)
Previous MI, n (%)	17 (37.8%)	1 (1.7%)
History of smoking, n (%)	27 (60%)	N/A
COPD, n (%)	6 (13.3%)	10 (16.7%)
Heart disease, n (%)	4 (8.9%)	5 (8.3%)
Creatinine level, mg/dL, mean ± SD	1.64 ± 1.93	1.3 ± 1.6
Ejection fraction, %mean ± SD	44.9 ± 13.2	52.27 ± 2.7
Ejection fraction <40, n (%)	18 (40%)	5 (8.3%)
PPM, n (%)	11 (24.4%)	5 (8.3%)
Mean preoperative STS score, %, mean ± SD	4.6% ± 5.6	3.60% ± 3.7

*SD*, Standard deviation; *BMI*, body mass index; *CVA*, cerebral vascular accident; *MI*, myocardial infarction; *N/A*, not applicable; *COPD*, chronic obstructive pulmonary disease; *PPM*, permanent pacemaker; *STS*, Society of Thoracic Surgeons.

### Intraoperative Outcomes

The mean operating room time was 260 minutes in the epicardial group and 237 minutes in the intracardiac group. Conversion to sternotomy was observed in 1 (2.2%) patient in the epicardial group and 1 (1.6%) of intracardiac group. Two (4.4%) patients in the epicardial group,required conversion to cardiopulmonary bypass from the intended off-pump approach.

Extubation in the operating room was performed in 11 (24.4%) patients in the epicardial group and 1 (1.6%) patient in the intracardiac group. Intraoperative blood transfusion was required in 4 (8.9%) patients in the epicardial group and 21 (35%) patients in the intracardiac group (Table 3).

**Table 3:** Intraoperative outcomes

Variables	Epicardial group (n = 45)	Intracardiac group (n = 60)
Mean OR time, min, mean ± SD	260 ± 101	237 ± 68
Conversion to bypass, n (%)	2 (4%)	N/A
Conversion to sternotomy, n (%)	1 (2%)	1 (1.6%)
Extubation in the OR, n (%)	11 (24.4%)	1 (1.6%)

*OR*, Operating room; *SD*, standard deviation; *N/A*, not applicable.

### Postoperative Outcomes

Extubation within 6 hours occurred in 18 (40%) patients in the epicardial group and 31 (51.6%) patients in the intracardiac group. Prolonged extubation ≥24 hours was observed in 2 (4.4%) patients in the epicardial group and 8 (13.3%) patients in the intracardiac group. Reintubation was required in 4 (6.7%) patients in the intracardiac group, whereas no patients required reintubation in the epicardial group.

Chest tube removal on postoperative day 1 was achieved in 24 (53.3%) patients in the epicardial group and 31 (51.6%) patients in the intracardiac group, with an average chest tube duration of 2.2 days in the epicardial group and 2.3 days in the intracardiac group.

Postoperative blood transfusion was necessary in 2 (4.4%) patients in the epicardial group and 20 (33.3%) patients in the intracardiac group. New-onset atrial fibrillation occurred in 5 (11.1%) patients in the epicardial group and 6 (10%) patients in the intracardiac group. Postoperative pneumonia was reported in 1 (2.2%) patient in the epicardial group and 2 (3.3%) patients in the intracardiac cases.

The mean ICU length of stay was 1.23 days in the epicardial group and 1.76 days in the intracardiac group. The mean hospital stay was 2.75 days in the epicardial group and 3.75 days in the intracardiac group.

There was no take-back for bleeding in the epicardial group, whereas 3 (5%) patients in the intracardiac group required reoperation for bleeding—all managed robotically without conversion to sternotomy. Thirty-day mortality was 2% (1/45) in the epicardial group and 1.6% (1/60) in the intracardiac group. Readmission within 30 days occurred in 4 (8.8%) of epicardial cases and 4 (6.6%) of intracardiac cases (Table 4).

**Table 4:** Postoperative outcomes

Variables	Epicardial group (n = 45)	Intracardiac group (n = 60)
Extubation within 6 h, n (%)	18 (40%)	31 (51.6%)
Extubation >24 h, n (%)	2 (4.4%)	8 (13.3%)
Reintubation, n (%)	0%	4 (6.7%)
CT removed on POD 1, n (%)	24 (53.3%)	31 (51.6%)
CT removal, d, mean ± SD	2.2 ± 2.5	2.3 ± 2.4
Postoperative blood transfusion, n (%)	2 (4.4%)	8 (13.3%)
New-onset atrial fibrillation, n (%)	5 (11.1%)	6 (10%)
Wound infection, n (%)	0%	0%
Pneumonia, n (%)	1 (2.2%)	2 (3.3%)
Postoperative ECMO, n (%)	1 (2.2%)	3 (5%)
Acute kidney injury, n (%)	1 (2.2%)	3 (5%)
ICU length of stay, d, mean ± SD	1.23 ± 0.64	1.76 ± 1.9
Hospital length of stay, d, mean ± SD	2.75 ± 2	3.75 ± 4
Take-back for bleeding, n (%)	0%	3 (5%)
30-d mortality, n (%)	1 (2%)	1 (1.6%)
Readmission within 30 d, n (%)	4 (8.8%)	4 (6.6%)

*CT*, Chest tube; *POD*, postoperative day; *SD*, standard deviation; *ECMO*, extracorporeal membrane oxygenation; *ICU*, intensive care unit.

### Midterm Follow-up

**Figure 1:**
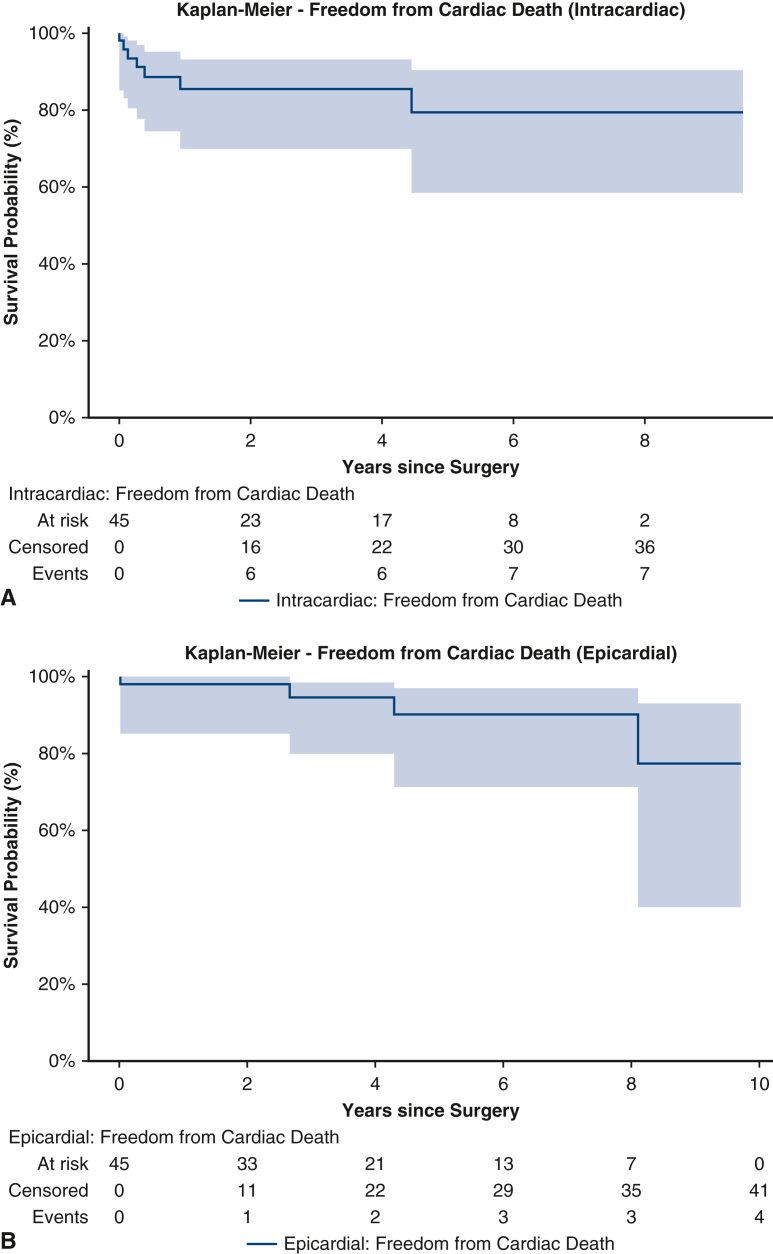
Kaplan-Meier survival curves for freedom from cardiac mortality in the (A) intracardiac and (B) epicardial groups. The x-axis represents years since surgery, and *shaded bands* represent 95% confidence intervals. The *tables* below each curve show the number of patients at risk at yearly intervals from 0 to 5 years.

At a mean follow-up of 32.4 months in the epicardial group and 36.2 months in the intracardiac group, all-cause mortality was 9 (20%) in the epicardial group and 7 (11.6%) in the intracardiac group. Cardiac-related mortality was 3 (6.7%) in the epicardial group and 4 (6.6%) in the intracardiac group. No patients were lost to early follow-up, and midterm data were available for more than 95% of patients (Table 5, Figure 1).

**Table 5:** Midterm follow-up outcomes

Variables	Epicardial group (n = 45)	Intracardiac group (n = 60)
Follow-up, mo, mean ± SD	32.4 ± 28	36.2 ± 33.5
All-cause mortality, n (%)	9 (20%)	7 (11.6%)
Cardiac-related mortality, n (%)	3 (6.7%)	4 (6.6%)
Freedom from MACCE, n (%)	42 (93.3%)	44 (73.3%)

*SD*, Standard deviation; *MACCE*, major adverse cardiac and cerebrovascular events.

## Discussion

This study demonstrates that robotic endoscopic cardiac surgery is feasible, safe, and effective in patients with previous cardiac surgery. All TECAB procedures included successful graft completion using intraoperative flow measurement tools. Similarly, mitral and valve procedures were confirmed intraoperatively and on follow-up as clinically successful.

Among 105 patients, low 30-day mortality (1.9%) and minimal conversion rates (1.9%) highlight the technical feasibility of the robotic approach, even in the complex reoperative setting. Patients undergoing epicardial procedures (TECAB, pericardiectomy, and left ventricular lead placement) had short ICU stays (1.23 days) and hospital stays (2.75 days), whereas patients undergoing intracardiac procedures (mitral, aortic, and tricuspid valve surgery) had similar favorable outcomes. The midterm follow-up (mean 36.2 months) revealed a cardiac-related mortality of 6%, with freedom from major adverse cardiac and cerebrovascular events of 93% (epicardial) and 73% (intracardiac).

These results suggest that cardiac reoperations can be performed using a robotic endoscopic approach with perioperative outcomes comparable with or better than traditional sternotomy-based reoperations, particularly in patients in whom avoiding sternal re-entry is highly beneficial.

One of the most critical advantages of robotic surgery in the reoperative setting is the potential to reduce the need for sternotomy in selected patients. The precision of robotic-assisted dissection allows for careful adhesiolysis and targeted exposure while reducing the risk of graft or cardiac injury. Previous studies, including those from our group,^17^ have emphasized the benefits of robotic-assisted adhesiolysis in preventing inadvertent damage to patent grafts and mediastinal structures. The historical risk of mortality for redo cardiac surgery via sternotomy is 4% to 7%, with increased morbidity from adhesiolysis and graft injury.^1^, ^2^, ^3^^,^^5^, ^6^, ^7^ Our findings of 1.9% early mortality and 1.9% sternotomy conversion rates reinforce previous reports suggesting that robotic surgery minimizes reentry complications and enhances surgical precision.

The ability to avoid a repeat sternotomy is particularly significant in patients with previous coronary artery bypass grafting, in whom injury to patent grafts poses a major risk.^2^^,^^19^ The ability to access the heart through a thoracoscopic approach minimizes manipulation of previous bypass grafts, preserving their function while reducing perioperative morbidity. Furthermore, previous studies have shown that redo sternotomy is associated with prolonged operative times because of the need for extensive dissection, whereas robotic surgery enables a more targeted approach with efficient adhesiolysis, reducing operative and bypass times in selected cases.

For redo coronary revascularization, techniques such as subxiphoid right gastroepiploic artery bypass have been used to avoid sternotomy.^14^, ^15^, ^16^ However, these approaches are often limited by anatomical constraints. Our results support previous findings that robotic TECAB enables safe and effective redo coronary surgery, aligning with studies showing excellent midterm survival and graft patency with robotic redo-TECAB.^10^^,^^13^^,^^17^^,^^20^

In addition to avoiding sternotomy, the ability to perform totally endoscopic TECAB in a redo setting maintains graft patency while minimizing morbidity. Previous studies have demonstrated that robotic-assisted revascularization provides comparable long-term graft patency with conventional approaches while reducing the need for prolonged mechanical ventilation and ICU stays. Given that many patients who undergo redo coronary artery bypass grafting have compromised sternal integrity from previous procedures, the ability to revascularize using a robotic approach is a significant advantage that can potentially enhance long-term outcomes by minimizing sternal complications. This approach can also allow the routine harvesting of an often previously unused RITA conduit. In this series of 31 redo TECAB procedures, 20 patients (65%) had a new RITA graft placed. Moreover, robotic TECAB enables multivessel bypass in selected cases, expanding the utility of robotic coronary surgery beyond single-vessel interventions.

For valve reoperations, minimally invasive techniques (eg, right thoracotomy) have been preferred over redo sternotomy.^9^^,^^21^ Our findings mirror those of large robotic mitral surgery series, where robotic reoperations demonstrated low mortality (0.6% to 2%) and reduced hospital stays. Our mean hospital stay (3.75 days for intracardiac procedures) is significantly lower than traditional redo valve surgery (∼7 days), reinforcing the benefits of the robotic endoscopic techniques.^2^^,^^19^

The success of reoperative robotic mitral and aortic valve procedures in this study further highlights the ability of the robotic platform to offer high-quality, durable repairs and replacements in a true minimally invasive fashion. In our experience, we believe that an endoscopic robotic approach using a very small working port (8 mm) can still be maintained in the reoperative setting as long as enough care and consideration are given to avoiding lung injury and meticulous adhesiolysis. Furthermore, the lateral approach to the mitral valve allows for much better visualization in the presence of intrapericardial adhesions or even a previous aortic valve prosthesis as we have previously reported.^22^ A special mention should be made about the importance of being versatile in the technique of cardiac arrest in the reoperative setting. For example, we have found hypothermic fibrillatory arrest to be a valuable option in selected patients with a patent internal thoracic artery graft undergoing reoperative robotic valve surgery. The ability to perform valve reoperations through a robotic endoscopic approach enables excellent visualization of the mitral and aortic valve apparatus while reducing the trauma associated with redo sternotomy. Our findings suggest that robotic valve reoperations can be performed by an experienced team with reproducible results and a favorable safety profile, supporting their increasing adoption in select high-risk redo patients.

### Clinical Implications

This study highlights the potential of robotic surgery in patients undergoing reoperative cardiac procedures. One of the major advantages of robotic TECAB is that it eliminates the need for sternotomy, reducing the risks associated with re-entry while preserving graft patency. Similarly, robotic mitral and aortic valve reoperations offer important benefits, including shorter hospital stays, less bleeding, and quicker recovery compared with traditional open approaches. The ability to perform precise adhesiolysis with robotic assistance allows for safer re-entry in patients with previous cardiac surgery, minimizing the risk of injuring critical structures. As cardiac surgeons become more and more comfortable with the robotic approach through dedicated training programs and wider adoption, they will join the ranks of other surgical subspecialists in leveraging this important technology with its superior visualization in avoiding some of the well-known drawbacks of traditional redo open surgery.^23^^,^^24^

In addition, beyond its benefits in the reoperative setting, robotic cardiac surgery has the potential to redefine the standard of care for selected high-risk patients. As more institutions develop dedicated robotic cardiac surgery programs, the experience gained in complex redo cases will continue to improve, ultimately leading to further reductions in morbidity and mortality. In addition, the ability to perform multiprocedure robotic interventions, such as combining mitral valve surgery with TECAB, represents an exciting frontier in minimally invasive cardiac surgery.

### Limitations

This study has several limitations that should be considered. It is a single-center retrospective study, meaning the results reflect the experience of one institution with a highly experienced robotic cardiac team and may not be applicable to all centers. Another limitation is the relatively small sample size of 105 patients, which, although one of the largest reported series on robotic reoperative cardiac surgery, still limits the generalizability of the findings. In addition, patient selection bias is a factor, because patients referred for robotic reoperations were likely carefully selected and treated by a highly experienced surgical team. Although the midterm outcomes are promising, long-term data beyond 5 years remain limited, and further studies are needed to assess durability and graft patency over time. Moving forward, randomized trials comparing robotic reoperations with conventional sternotomy-based approaches will be necessary to fully understand the long-term benefits and ideal patient selection for robotic redo cardiac surgery.

Another limitation of this study is the lack of direct comparison with alternative minimally invasive techniques, such as right thoracotomy approaches. Although our results demonstrate favorable outcomes with robotic reoperations, a head-to-head comparison with other minimally invasive options would provide a clearer perspective on the advantages and disadvantages of each approach.

## Conclusions

This study confirms that robotic endoscopic cardiac surgery in reoperative patients is feasible, safe, and effective when performed by an experienced team. By avoiding sternotomy, reducing perioperative morbidity, and achieving midterm survival rates comparable with or better than traditional approaches, robotic surgery represents a viable option for carefully selected redo patients.

The low mortality and excellent 30-day and midterm outcomes suggest that robotic approaches can provide durable outcomes in the reoperative setting. Robotic-assisted cardiac surgery offers a minimally invasive approach that preserves previous surgical grafts, reduces complications, and enhances recovery for patients requiring redo operations.

These favorable results compare very well with those reported in large multicenter series of reoperative cardiac surgery. In a recent propensity-matched analysis by Bianco and colleagues^25^ of more than 3000 patients, 30-day and 1-year mortality rates for reoperative cardiac surgery were reported as 8.1% and 16.4%, respectively, with a 14.1% reoperation rate and an average ICU stay exceeding 98 hours. By contrast, our series—which is among the largest to date using a totally robotic endoscopic approach—demonstrates markedly lower 30-day mortality (1.9%), reoperation for bleeding (2.9%), and ICU stay (1.6 days), underscoring the potential benefits of a minimally invasive approach in the reoperative setting. Moving forward, future studies should focus on long-term outcomes, patient selection optimization, and advancements in robotic technology to further improve the safety and efficacy of robotic cardiac reoperations.

### Webcast

You can watch a Webcast of this AATS meeting presentation by going to: https://www.aats.org/resources/robotic-endoscopic-cardiac-sur-9906.

**Figure:**
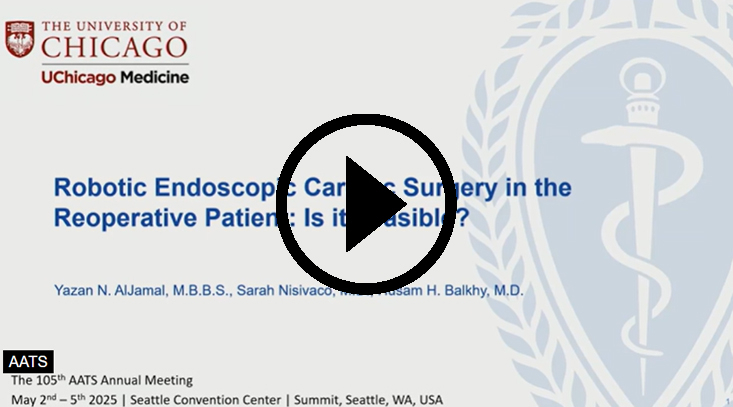

### Audio

You can listen to the discussion audio of this article by going to the supplementary material section below.

## Conflict of Interest Statement

H.B. is a proctor for Intuitive. All other authors reported no conflicts of interest.

The *Journal* policy requires editors and reviewers to disclose conflicts of interest and to decline handling or reviewing manuscripts for which they may have a conflict of interest. The editors and reviewers of this article have no conflicts of interest.

## Supplementary Data

### Audio

Audio 1 This is an audio recording of the presentation of the associated abstract and discussion. The abstract presentation ends at 9:18 and the discussion begins at 9:23.

Video 1 Robotic endoscopic cardiac surgery in the reoperative patient. Video available at: https://www.jtcvs.org/article/S2950-6050(25)00038-5/fulltext.
