# Supplementary figures and images for: Robotic endoscopic cardiac surgery in the reoperative patient: Is it feasible?
Source: JTCVS Struct Endovasc. 2025 Sep 17;8:100079. doi: 10.1016/j.xjse.2025.100079 (PMC13244753; doi:10.1016/j.xjse.2025.100079)

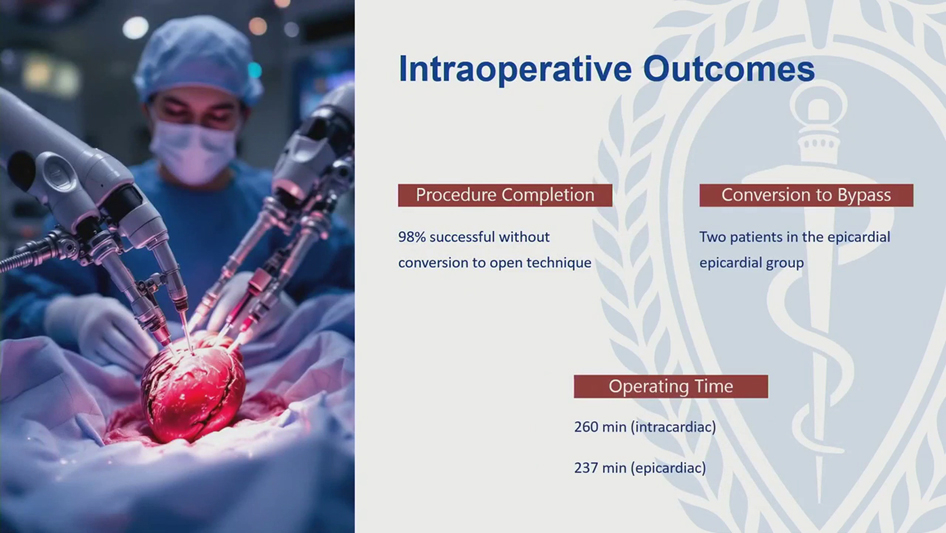

Supplement: Video 1 — Robotic endoscopic cardiac surgery in the reoperative patient. Video available at: https://www.jtcvs.org/article/S2950-6050(25)00038-5/fulltext. [file fx3.jpg]
